# Accelerating tobacco control at the national level with the Smoke-free Generation movement in the Netherlands

**DOI:** 10.1038/s41533-022-00321-8

**Published:** 2022-12-23

**Authors:** Marc C. Willemsen, Jasper V. Been

**Affiliations:** 1 Trimbos Institute, Department for Tobacco Control, Utrecht, the Netherlands; 2 Maastricht University, Department of Health Promotion, Maastricht, the Netherlands; 3 Division of Neonatology, Department of Paediatrics, Erasmus MC Sophia Children’s Hospital, University Medical Centre Rotterdam, Rotterdam, the Netherlands; 4 Department of Public Health, Erasmus MC, University Medical Centre Rotterdam, Rotterdam, the Netherlands

**Keywords:** Health policy, Public health, Paediatrics

## Abstract

The Netherlands has moved towards the forefront of tobacco control in Europe, after having implemented important tobacco control measures in 2020 and 2021, which included higher tobacco taxation, plain packaging of tobacco products, a partial point of sale tobacco display ban, smoking ban on school grounds, and other smoking restrictions. We examined the factors contributing to these successes, focussing on the network of tobacco control advocacy organisations and the process of agenda-setting. Crucial determining factors were stricter adherence to Article 5.3 FCTC, which prevents government to consult tobacco industry, and the genesis of a ‘Smoke-free Generation’ movement in the wider society, initiated by the three main national charities (Lung Foundation Netherlands, Dutch Heart Foundation, Dutch Cancer Society). The Smoke-free Generation concept proved to be a highly attractive unifying strategy for national en local policy makers and Dutch society. As a result, the Dutch government was able to start a process of strengthening tobacco control policy through drafting and implementing a National Prevention Agreement, which aims at a tobacco control endgame goal of less than 5% smokers in 2040. Between 2019 and 2020 smoking rates dropped from 21.7% to 20.2%. The Dutch experience can provide inspiration for countries where tobacco control is still lagging behind. It also illustrates that continued vigilance is needed, since the most recent government change was associated with a hampering of further reduction of the proportion of smokers and a temporary drop in attention to tobacco control from the central government.

## Introduction

In most countries in the world, smoking rates are going down gradually, albeit slowly. The World Health Organization (WHO) estimated that the age-standardized prevalence of tobacco smoking has decreased on average 0.4 percentage points per year since the beginning of the 21st century^1^. However, countries that implemented key WHO Framework Convention on Tobacco Control (FCTC) measures at the highest level (i.e., taxation, smoke-free policies, warning labels, advertising, promotion and sponsorship bans, and cessation programmes) were able to decrease their smoking rates faster^2^. A recent study calculated that if all countries had implemented key tobacco control WHO’s FCTC measures to the strictest level in 2017, there would have been about 100 million fewer smokers^3^.

A second important aim of tobacco control measures is to reduce exposure to second-hand smoke among non-smokers. While in 2007 only 3% of people worldwide were protected by smoke-free policies at the highest level, this had increased to 24% in 2020^4^. Introducing smoke-free legislation leads to improved health outcomes for populations, with strongest evidence in reduced admissions for acute coronary syndrome^5^. In addition, implementation of comprehensive smoke-free laws has been associated with benefits to child health, including reductions in rates of preterm birth and fewer hospital attendances for asthma exacerbations^6^.

Despite the paramount importance to have a strong and comprehensive national tobacco control policy, European countries vary widely in terms of how many policy measures have been adopted and implemented^7^. The Tobacco Control Scale (TCS) monitors the implementation of tobacco control policies at country level across Europe. The Netherlands is one of the European countries which performed moderately well in 2019, ranking 14th of 36 European countries in terms of the comprehensiveness of tobacco control policy in 2019^7^. Main measures that had been implemented were a partial smoking ban for public places and workplaces, a tobacco advertising and promotion ban (but not yet a display ban at points of sale), pictorial health warnings on cigarette packs (but not yet plain packaging)^7^. Notably the Dutch per capita budget allocated to tobacco control has been low relative to other European countries as have cigarette prices when taking into account purchasing power standards^7^. TCS scores are calculated every three years. Ranking for the Netherlands was much higher in 2022, because various important tobacco control measures have been implemented by the government since 2019, including a tax increase of one euro for a pack of cigarettes, plain packaging for tobacco products, prohibiting designated smoking rooms in bars, restaurants and other workplaces, smoke-free school premises, bans on the display of tobacco products at point-of-sale, and improvements in how smoking cessation support is covered by the national health insurance system^8^.

These recently adopted policy measures were made possible because the tobacco issue had been put firmly on the government’s policy agenda in the preceding years, following a process of agenda-setting which started around 2014. This process is described in this paper. As a theoretical framework we used Willemsen’s conceptual framework for understanding tobacco control policy making^9^. This framework builds on the major strands of thought in public policy science regarding policy change^10–14^ and applies these to the tobacco control policy field. According to the framework, adoption of strong tobacco control policy by a government is contingent on sufficient political support in parliament and cabinet. Such support is the direct result of (1) relative effectiveness of lobbying practices by tobacco control advocacy groups and how effective these groups are in building supportive networks, and (2) beneficial changes in the problem definition (issue framing) and agenda-setting by advocacy groups^9^. The present perspective article aims to explain how these two important factors (networks and agenda setting) contributed to understanding recent successes in tobacco control in the Netherlands.

### Networks: building a strong Dutch tobacco control advocacy coalition

Around 2009, the political climate for tobacco control in the Netherlands was unfavourable, with little support in society for stronger tobacco control. At the time, The Netherlands had the lowest percentage (22%) of smokers who have a ‘negative’ or ‘very negative’ opinion regarding smoking compared to 16 countries surveyed^15^. In 2010, a newly appointed government rolled out a conservative political agenda of less governmental influence and budget cuts, which had dramatic negative consequences for tobacco control policy: existing smoke-free laws were weakened by allowing smoking in small bars again^16^ and most of the government’s tobacco control operations were closed down^17^. STIVORO, which had been the national coordinating tobacco control organisation since 1975, responsible for implementing tobacco control interventions such as educational campaigns and providing smoking cessation support to smokers, was disbanded. A more extensive account of why Dutch tobacco control was lagging behind other developed countries can be found in a previous publication of the first author^9^.

Historically, industry lobby in the Netherlands was very influential and stable due to high-positioned contacts in the Christian-Democratic party which was always in the centre of power. It was normal practice that ministers, supported by top civil servants, met in person with the tobacco industry. The contact between industry and the government’s bureaucracy was challenged after the government ratified WHO’s FCTC treaty in 2005. While the tobacco industry attempted to keep its communication lines open, referring to cherished Dutch traditions of consultation, this was countered by effective activities by the tobacco control community. In 2013, the Youth Smoking Prevention Foundation began to name and shame people associated with the industry through their website ‘www.tabaknee.nl’ and set out a programme of revealing industry tactics in the media, resulting in more societal pressure on the government to adhere to Article 5.3 FCTC. Article 5.3 states: “In setting and implementing their public health policies with respect to tobacco control, Parties shall act to protect these policies from commercial and other vested interests of the tobacco industry in accordance with national law”^18^.

In 2012, with a new government and a health minister from the Labour Party, the tension between government and health organizations over tobacco control had lessened. However, despite the Netherlands having ratified the FCTC in 2005, implementation of FCTC measures was still very poor^19^. There was a strong sense of urgency within the tobacco control community to take action. For several decades, The Dutch Cancer Society, Dutch Heart Foundation and Lung Foundation Netherlands had been strong champions for tougher tobacco control, working together with the government through their mutual financial support for the—now defunct—STIVORO. The three charities realized that they had to continue to work together, but not anymore in tandem with the government, which had let them down. They decided to bring their tobacco control activities together and founded a new organization to coordinate this^9^. They also understood that they had to broaden the fight for tobacco control by including as many societal organisations as possible into one comprehensive tobacco control coalition. Coordination was taken up by a newly formed organization in 2014, which was called Dutch Alliance for a Smoke-free Society (*Alliantie Nederland Rookvrij*; ANR).

The ANR can best be described as a ‘position-oriented coalition’, an autonomous advocacy group, whose “existence is based on achieving a list of legislative objectives that represents the broadest possible agreement”^20^. ANR formulated three strategic goals: (1) first create societal and then political support for a smoke-free society, (2) promote feasible and evidence-based measures that follow directly from the WHO’s FCTC treaty, and (3) establish partnerships with as many societal stakeholders as possible. Individual organizations could become members by formally endorsing the aims and general strategy, while day-to-day and strategic decisions were made by the coalition coordinator of the ANR bureau. According to their capacities and fields of interests, coalition partners were allocated to subthemes, such as making specific areas smoke-free or improving smoking-cessation support in medical settings, as long as they contributed to the overall strategy.

### Agenda setting: the Smoke-free Generation campaign

**Fig. 1:**
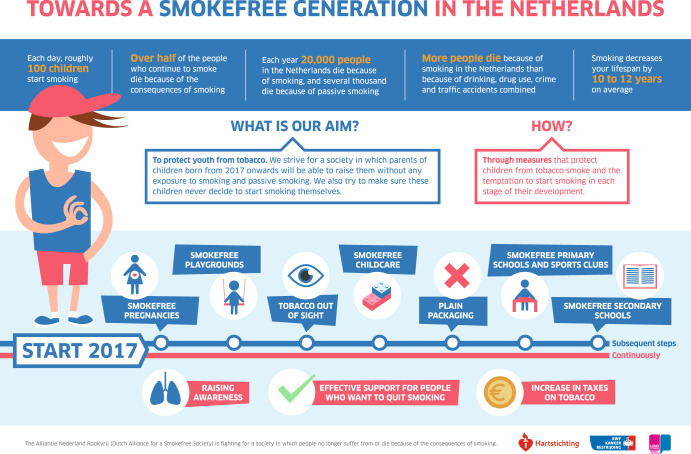
Roadmap towards a Smoke-free Generation in the Netherlands. Infographic developed by Dutch Alliance for a Smokefree Society, currently ‘Health Funds for Smoke-free’ (*Gezondheidsfondsen voor Rookvrij*; GvRV); reprinted with permission.

ANR understood that the Dutch political context is historically characterised by a broad dislike of paternalism in society. ANR identified the issue frame of protecting children against smoking and the temptation of tobacco as an overarching theme that is acceptable to the Dutch public and to politicians, including to those in the liberal-conservative political spectrum. Research among Dutch adults has indeed shown that emphasizing the need to protect children against tobacco enhances support for tobacco control policies^21^. The strategy was “to allow parents to raise their children free from exposure to tobacco smoke and from the temptation to start smoking; so that all children who are born from 2017 onwards, will choose to never start smoking”^22^, aiming for a smoke-free generation by 2035^23^. The ‘protection of children’ frame was chosen as an ‘umbrella’ for all ANR activities. The idea was that in the years to come, more and more venues where children may be confronted with tobacco smoking will become totally smoke- or tobacco-free. This was illustrated by a ‘Roadmap towards a Smoke-free Generation’ developed by ANR (Fig. 1). The roadmap follows the lifeline of a child born in 2017, and points out which smoke-free measures should be taken by the national government, municipalities and (civil) society to fully protect youth from exposure to tobacco in each stage of their development, starting with smoke-free pregnancies, followed by smoke-free playgrounds and other environments that children frequent^23^. The roadmap also indicates the three main pillars of effective tobacco control: raising awareness through campaigns, providing support for those who want to quit smoking, and increasing the price of tobacco through taxation.

The campaign started in November 2015, when ANR launched a series of television commercials, featuring young children talking about their dislike of when grownups smoke. The voice-over said: “Allow children to grow up smoke-free. You can help. Join us on the road to a Smoke-free Generation.” By using a step-by-step approach, more and more societal partners were motivated to contribute and join the alliance^22^. Through a coordinated interplay between mass communication about the Smoke-free Generation campaign and an active political lobby with stakeholders who speak out and support the Smoke-free Generation, the Smoke-free Generation campaign received wide support from the general public and turned into a ‘Smoke-free Generation movement’.

In 2016, the Youth Smoking Prevention Foundation took the State to court for violating Article 5.3. A break-through came when the government gave in and began to adhere to its obligations not to consult the industry under Article 5.3 FCTC^24^. The tobacco industry lost its former insider position and the balance between the tobacco control movement and the tobacco industry coalition shifted much more in favour of the health network. With its Smoke-free Generation movement, ANR succeeded in binding a large and diverse set of stakeholders, including municipalities and medical organisations to the ‘Smoke-free Generation’ aspiration. Every year, ANR hands out Smoke-free Generation Awards to recognise the work done by municipalities and their representatives to promote the smoke-free message. For example, in 2018 a total of 58 initiatives were nominated in six categories, of which six received a national award^25^. Within a year of its conception, ~40 partner organizations had joined ANR. The movement grew to more than 70 partners in July 2017^26^, and in 2018, the alliance had received endorsement from more than 100 organisations^23^. Partners were medical organisations (representing nurses, various specialists and general physicians), several universities, many municipalities seeking inspiration for local tobacco control initiatives and organisations that provided smoking cessation counselling to smokers.”

**Fig. 2:**
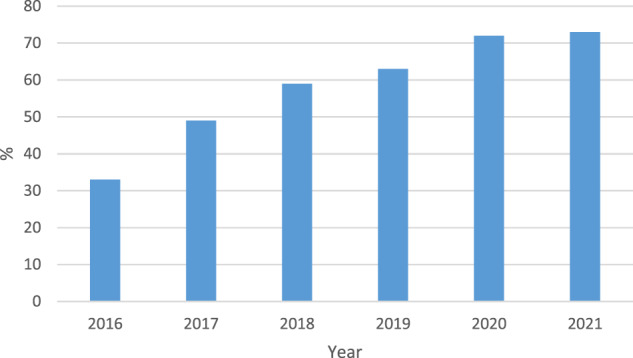
Familiarity with the concept ‘Smoke-free Generation’. Proportion of Dutch respondents (annual representative sample ≈1000 adults) acknowledging familiarity with the ‘Smoke-free Generation’ concept. Data collected by Kantar Public on behalf of ‘Health Funds for Smoke-free’ (*Gezondheidsfondsen voor Rookvrij*; GvRV).

Societal exposure to the Smoke-free Generation movement was assessed by Kantar Public from 2016 onwards. A representative annual sample of over 1000 Dutch adults (aged 18 years or older) were asked if they were familiar with the concept of the ‘Smoke-free Generation’. The percentages who answered affirmative increased from 33% in 2016 to 73% in 2021 (see Fig. 2)^27^. Smokers (80%) were more familiar with the movement than never-smokers (71%) and ex-smokers (70%)^27^. In addition, 73% of Dutch adults said the Smoke-free Generation “appeals to me”, and 75% found it important that “our society dedicates to a Smoke-free Generation”^27^. The vast majority of the population, including most smokers, supported smoke-free policies for places frequented by children, such as school grounds, private cars, playgrounds, children’s day care facilities, and petting zoos^27^. In 2019, the WHO presented the World No Tobacco Day Award to the ANR for the Smoke-free Generation campaign.

### The Network Netherlands Smoke-free

Because the increasing number of organisations joining the alliance became too large to centrally coordinate and support, the three charities reorganized their activities in 2022. ANR was renamed into ‘Health Funds for Smoke-free’ (*Gezondheidsfondsen voor Rookvrij*; GvRV). It continued its mission to coordinate and support the Smoke-free Generation movement in Dutch society and to lobby for more effective tobacco control policy^28^. The strategy to centrally coordinate a large number of individual societal organisations within one alliance was replaced by an open network approach, where independent tobacco control ‘coalitions’ (comprising of multiple individual organisations) work around a specific theme while sharing the mission of wanting to contribute to a smoke-free society. They share a communication platform, which is called ‘Network Netherlands Smoke-free’^29^. To date, the network consists of ten local coalitions (around the theme of smoke-free environments), two smoke-free sport coalitions, four health care coalitions, a smoke-free schools coalition, and a generic advocacy (lobby) coalition.

### The National Prevention Agreement (NPA)

In October 2017, a new government came to power, consisting of a four-party coalition comprising two conservative parties (VVD, CDA) and two progressive parties (D66 and CU). ANR’s lobby succeeded in having the ‘Smoke-free Generation’ featured in the coalition agreement that was the starting point of the new cabinet’s policy agenda: “We support the goal of creating a Smoke-free Generation and will therefore increase excise duty on tobacco”^30^. The agreement further stated that “A national preventive healthcare agreement will be concluded with patient organisations, care providers, health insurers, municipalities, sports clubs and associations, businesses, and civil society organisations. The focus of the agreement will be on tackling smoking and obesity.”

Paul Blokhuis, the new State-Secretary for Health, and ultimately responsible for managing this national prevention agreement, turned out to be a strong champion for tobacco control. This provided the long-awaited window of opportunity to advance tobacco control. A National Prevention Agreement (NPA) was signed by over 70 organisations in November 2018, and included policy intentions to address the key preventable drivers of adverse health outcomes: excessive alcohol use, overweight and obesity, and tobacco use. For tobacco, the stated goals were to have—by 2040—less than 5% smokers, 0% smoking children, and 0% pregnant women who smoke^31^.

Through a process of ‘thematic round tables’, the government sought concrete commitment from civil society organisations. In line with Article 5.3 FCTC, tobacco industry representatives were not allowed to sit at the table, or be involved in the process^32^. This made it more difficult for the industry to lobby, but not impossible, since they continued to approach parliamentarians and ministries^24^. At the ‘tobacco theme table’ 31 organisations, including GvRV, contributed and became partners to the Agreement. The tobacco industry was excluded from these meetings. In November 2018, the NPA was signed. It included an extensive package of policy measures^31^, most of which were implemented in 2020. The main regulatory measures were:

- Higher tobacco taxes resulting in an increase of the price of a pack of 20 cigarettes by 1 euro and a pack of roll-your-own tobacco by 2.5 euros (effective April 1, 2020).
- Existing smoke-free regulations extended to also cover electronic cigarettes (effective July 1, 2020).
- Ban on the display of tobacco products at the points-of-sale (effective July 1, 2020 in supermarkets; per January 1, 2021 extended to all other selling venues, with the exception of tobacco specialty stores).
- Ban on smoking on school premises (including universities and university hospitals; effective August 1, 2020).
- Plain packaging for tobacco products (effective October 1, 2020).
- End to dedicated smoking rooms in public buildings (effective July 1, 2021) and the private sector (effective January 1, 2022).
- Ban on tobacco vending machines (effective January 1, 2022).

It must be noted that the package of policy measures could have been stronger, in the full absence of tobacco industry lobbying. Most importantly, due to a tobacco industry lobby directed at the parliament, taxation was limited to one increase in 2020 (despite yearly tax hikes) and the government agreed that research first had to be carried out to examine any border effects of the price increase, before it would decide on further tax increases^33^.

The government committed to support municipalities financially to draft local Smoke-free Generation policies and financed yearly smoking cessation mass media campaigns (‘PUUR Rookvrij’ and Stoptober). In addition to the national policy measures, the Agreement included a list of actions and measures that societal organizations were committed to take to realize smoke-free environments bottom-up. These organizations included petting zoos, children’s playgrounds, children day care centres, sports clubs (expected to be smoke-free by 2025), general hospitals, and other health care institutes (including addiction and mental health institutes). The National Institute for Health and Environment (RIVM) was tasked to monitor progress of the Prevention Agreement commitments through annual progress reports and a 4-year review of progress in primary outcomes towards the ambitions for 2040. The first of these 4-year reports is expected in 2023.

**Fig. 3:**
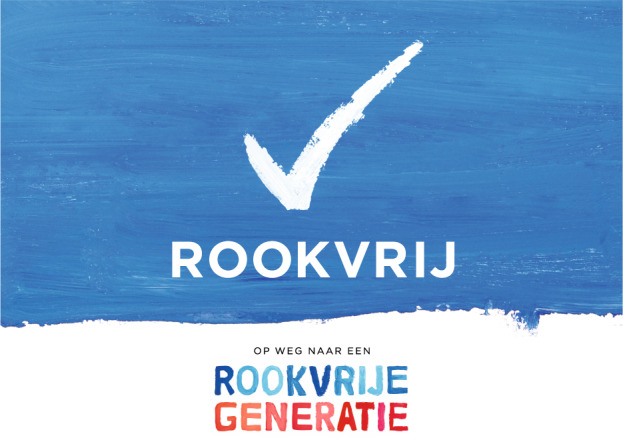
Smoke-free Generation sign. Uniform design of a sign to depict an indoor or outdoor area as smoke-free. Translation: ‘Smoke-free; Moving towards a Smoke-free Generation’. ‘Health Funds for Smoke-free’ (*Gezondheidsfondsen voor Rookvrij*; GvRV); reprinted with permission.

In 2021, 55% of Dutch municipalities actively supported smoke-free activities and did so under the banner of the ‘Smoke-free Generation’ movement^34^. The number of sports clubs with an outdoor smoke-free policy increased from 0.3% in 2016 to 26.4% in 2020, with field hockey and korfball clubs and clubs with relatively many youth members taking the lead^35^. By 2021, almost 2000 outdoor sports associations and about 8 out of 10 recreational youth facilities (playgrounds, petting zoos) were self-declared smoke-free^36^. GvRV developed and distributed signs, that organisations throughout the Netherlands use to indicate that they are smoke-free and support the Smoke-free Generation movement (Fig. 3).

Following the implementation of the NPA measures in 2020, the national smoking rate dropped from 21.7% in 2019 to 20.2% in 2020^37^.

## Discussion

Major tobacco control policy changes only happen when a window of opportunity opens and three conditions are simultaneously in place: sufficient attention to the tobacco problem, consensus about the solutions to the problem, and political will^13^. In the Netherlands, this window opened when a new State-Secretary for Health responsible for tobacco control came to office in 2017. State-secretary Paul Blokhuis was personally motivated to combat smoking. In addition to political will, there had been created wide-spread consensus on how to proceed with tobacco control policy and the issue had been put firmly on the political agenda. We showed that this was the direct result of an effective lobby by ANR, which succeeded in building a broad supportive societal anti-tobacco network, and of its decision to define the problem of tobacco control in terms of protecting youth and aiming for a Smoke-free Generation (issue framing). Smoke-free Generation ambitions became central to the new government’s National Prevention Agreement, which was a long-term commitment from the government to tackle smoking, aiming for a smoke-free society by 2040.

In the preceding years (2015–2017), ANR had paved the way by bringing together a large number of societal organisations under the umbrella of the Smoke-free Generation movement. This included national advocacy groups, including Clean Air Netherlands (CAN), which had taken the government to court over smoke-free bars and the Youth Smoking Prevention Foundation which had forced the government through legal action to adhere to Article 5.3 FCTC. The latter was important to assure that the tobacco industry’s ability to lobby against stronger tobacco control measures was reduced. It was then relatively easy for the State-Secretary to include tobacco control policy legal measures into the new NPA, trusting that there was broad societal and political support for these measures.

Governments’ longer-term tobacco control policy commitments are fragile. The year 2021 was characterised by a very long interim period between governments. It took nine months after the elections from 17 March 2021 before a new cabinet was installed. In that period, national tobacco control policy making was virtually non-existent and hardly any new measures were implemented. In parallel, the year-to-year reduction in smoking prevalence came to a standstill. In 2021, 20.6% of the adult population smoked^37^, which suggests an increase instead of a reduction in the number of smokers, as the percentage of smokers was 20.2% in 2020. However, some of the smoking uptake must be attributed to the COVID-19 pandemic. In the Netherlands, the majority of smokers reported to smoke more due to the COVID crisis rather than less^38^. Although there now exists a long-term commitment from the Dutch government to tackle smoking, continued vigilance is called for during periods when governments shift in power and the tobacco industry continues to lobby for policy weakening. It is slightly worrying that the Prevention Agreement is mentioned in the new coalition agreement but tobacco control and Smoke-free Generation are not explicitly referred to^39^. The budget for tobacco control has not been increased and only one further increase in tobacco taxation is mentioned in the coalition agreement. Without intensifying tobacco control policy, it will become difficult to reach the goal of <5% smokers in 2040.

The genesis of a ‘Smoke-free Generation’ movement in the Netherlands, initiated by the three main national charities (Lung Foundation Netherlands, Dutch Heart Foundation, Dutch Cancer Society) proved to be a unifying strategy for national en local policy makers to work together to advance tobacco control, with broad support from politicians. However, the final choice of which policy measures to implement remains subject to ideological preferences of the politicians in power.
